# JiffyNet: a web-based instant protein network modeler for newly sequenced species

**DOI:** 10.1093/nar/gkt419

**Published:** 2013-05-17

**Authors:** Eiru Kim, Hanhae Kim, Insuk Lee

**Affiliations:** Department of Biotechnology, College of Life Science and Biotechnology, Yonsei University, Seoul, 120–749 Korea

## Abstract

Revolutionary DNA sequencing technology has enabled affordable genome sequencing for numerous species. Thousands of species already have completely decoded genomes, and tens of thousands more are in progress. Naturally, parallel expansion of the functional parts list library is anticipated, yet genome-level understanding of function also requires maps of functional relationships, such as functional protein networks. Such networks have been constructed for many sequenced species including common model organisms. Nevertheless, the majority of species with sequenced genomes still have no protein network models available. Moreover, biologists might want to obtain protein networks for their species of interest on completion of the genome projects. Therefore, there is high demand for accessible means to automatically construct genome-scale protein networks based on sequence information from genome projects only. Here, we present a public web server, JiffyNet, specifically designed to instantly construct genome-scale protein networks based on associalogs (functional associations transferred from a template network by orthology) for a query species with only protein sequences provided. Assessment of the networks by JiffyNet demonstrated generally high predictive ability for pathway annotations. Furthermore, JiffyNet provides network visualization and analysis pages for wide variety of molecular concepts to facilitate network-guided hypothesis generation. JiffyNet is freely accessible at http://www.jiffynet.org.

## INTRODUCTION

With advanced DNA sequencing and genome assembly technology, >4000 completely decoded genomes have already been deposited in public databases as reported by Genome Online Database (http://www.genomesonline.org) of January 2013. This already impressive number has recently been growing even faster due to the revolution in next-generation sequencing technology. The same database also reported ∼15 000 more genome projects in progress; thus tens of thousands of completely sequenced species will be publicly available in the next few years. In addition, biologists recently set a new goal of sequencing genomes for 10 000 vertebrate species (Genome 10K Project, http://genome10k.soe.ucsc.edu/), and 5000 insect and related arthropod species (i5k initiative) in next 5 years (1). Sequence analysis algorithms can deliver fairly accurate gene or protein models, thus providing functional parts lists for numerous species along with their sequenced genomes. However, system-level functional understanding of organism traits also requires maps of functional relationships between the molecular parts, such as genome-scale protein networks (2).

Experimental approaches for constructing genome-scale protein networks are expensive and time-consuming. Consequently, only few model organisms have been tackled by genome-wide screens for protein interactions to date. In contrast, computational approaches have proven efficient for modeling relationships between proteins in many species. For example, if two proteins interact in one species, the orthologous pair of proteins in other species is also likely to interact. The protein interaction inferred by orthology has been coined interolog (orthologous interaction) (3), and this approach has been adapted to construct protein networks for many species, particularly for those that lack experimentally determined protein interactions. The basic concept of interolog has been extended to functional association, where if two proteins are functionally associated in a species, their orthologous proteins in other species are also likely to associate, and the inferred association is dubbed ‘associalog’ (orthologous association) (4). Because two proteins may operate in the same pathways with no physical interaction, many functional associations are not necessarily supported by physical interactions. Therefore, protein networks based on functional associations are generally more complete than those based on physical interactions.

The algorithmic simplicity of orthology-based inference of protein relationships may allow automating the whole process of network construction. Biologists may lack the required skills and knowledge in network biology, but still want to obtain protein networks for their species of interest on completion of genome sequencing. Therefore, we developed JiffyNet, a web-based automated pipeline of orthology-based network modeling of newly sequenced species. The quality of orthology-based networks is largely determined by accuracy as well as completeness of the template networks, from which associalogs for the new network are inferred. JiffyNet uses highly accurate genome-scale template protein networks, compiled from various independent lines of evidence using Bayesian statistics framework (5,6). The current version of JiffyNet provides template networks for six diverse species, spanning from *Escherichia coli* to human.

The template networks in JiffyNet provide an edge weight score (likelihood of functional association), facilitating robust hypothesis generation using “guilt-by-association” principles. We retain the robustness of probabilistic edge scores by combining likelihood scores of template network edges with orthology-related scores. For further functional interrogation of the query species protein network, JiffyNet also provides subnetwork visualization and analysis pages for wide variety of molecular concepts (7) by Gene Ontology (GO) (8) and Kyoto Encyclopedia of Genes and Genomes (KEGG) pathway (9) definitions.

BIANA Interolog Prediction Server (BIPS, http://sbi.imim.es/BIPS.php) is another public webserver capable of conducting orthology-based network modeling (10). However, JiffyNet substantially differs from BIPS as follows: (i) JiffyNet was specifically designed to infer whole protein networks, whereas BIPS deduces individual protein–protein interactions; (ii) JiffyNet is based on associalogs, whereas BIPS is based on interologs; (iii) JiffyNet uses genome-scale protein networks as template relationship sets unlike BIPS, which uses all protein–protein interactions derived from multiple public databases; (iv) JiffyNet provides confident scores for inferred network edges not available from BIPS; and (v) JiffyNet provides network visualization and analysis services absent from BIPS.

## DESCRIPTION OF JIFFYNET

**Figure 1.**
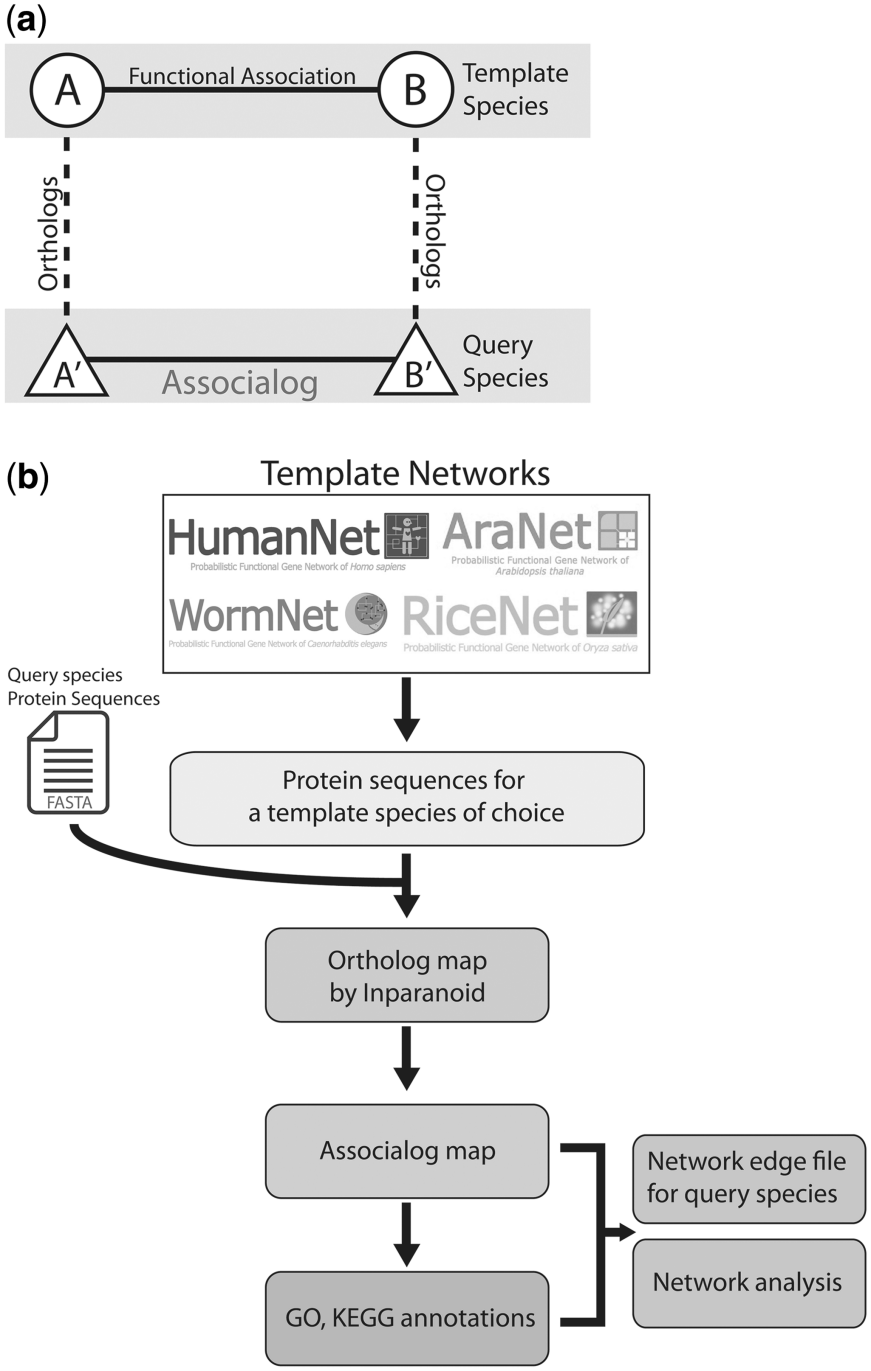
(**a**) Illustration of associalog, which is an inferred functional association between protein A′ and B′ in a query species. A′ and B′ are orthologs of template species protein A and B, respectively. (**b**) Schematic summary of network construction and analysis processes implemented in JiffyNet web server.

JiffyNet builds protein networks using associalogs (Figure 1a) based on template networks made of functional associations between proteins. Network edge representation by functional perspectives enhances network completeness because its broad definition of relationship includes diverse types of molecular interactions other than just physical interaction (5). Indeed, all the template networks of JiffyNet cover large portions of the template species coding genomes (Table 1) and potentially do so for query species.

**Table 1.** Template networks for the JiffyNet webserver

Template network	Template species	No. of proteins (coverage of coding genome)	No. of functional associations
EcoliNet (draft version)	*E. coli*	4117 (99%)	120 510
YeastNet (version 2)	*S. cerevisiae*	5483 (95%)	102 803
WormNet (version 2)	*C. elegans*	15 139 (75%)	999 367
HumanNet (version 1)	*H. sapiens*	16 242 (87%)	476 399
AraNet (version 1)	*A. thaliana*	19 647 (73%)	1 062 222
RiceNet (version 1)	*O. sativa*	18 377 (45%)	588 221

The overall process implemented in the JiffyNet system is summarized in Figure 1b. The current version of JiffyNet is based on six template networks from species of bacteria, fungi, animals and plants: EcoliNet (unpublished version), YeastNet (11), WormNet (12), HumanNet (13), AraNet (14) and RiceNet (15) for *E. coli, Saccharomyces cerevisiae*, *Caenorhabditis elegans*, *Homo sapiens*, *Arabidopsis thaliana* and *Oryza sativa*, respectively (Table 1). The quality of the template networks has been demonstrated by various computational as well as experimental validations as previously reported. We recommend users select the template species closely related to their query species. For example, EcoliNet is recommended for bacteria, YeastNet for fungi, WormNet or HumanNet for animals and AraNet or RiceNet for plants.

JiffyNet requires input protein sequences of a query species as a FASTA file. After uploading an input sequence file and selecting an appropriate template network, the user starts the automated network construction process by clicking ‘SEND’ button. In this step, the user may also submit an optional email address if email notification is preferred on completion of the submitted job. The action of query submission prompts a new message page showing an assigned ‘Query ID’. The user needs to bookmark this page or copy the Query ID to check status of the submitted job or access the final results later. Due to the generally long calculation time for ortholog mapping, submitted queries may take up to several days to finish, particularly between templates and query species with a large number of proteins. Rice, for example, has >40 000 protein coding genes. A table listing estimates of the computational time required for a given query is available on the tutorial page of the JiffyNet web server. Moreover, the capacity of the current JiffyNet server is running up to five queries at a time. Thus, one may need to wait for next available service in queue. For such situations, JiffyNet allows users to check status (Queued, Running or Finished) of jobs in the middle of processes.

JiffyNet first maps orthologous relationships between all input query proteins and all proteins of the selected template species using the INPARANOID algorithm (http://inparanoid.sbc.su.se) (16). This algorithm enables highly sensitive ortholog mapping by considering recent paralogs (inparalogs) as co-orthologs. Compared with other algorithms, INPARANOID best achieves the optimal balance between sensitivity and specificity (17).

The edge weight score, or log likelihood score (LLS) (5), of the template networks is the log-scaled likelihood of functional association between two linked proteins. If the likelihood equals the value expected due to chance alone, the LLS will be zero. By contrast, if the LLS is four, the likelihood that two proteins associate with one another is ∼55 times higher than the value expected by chance alone. The LLSs of template networks are transferred to their corresponding associalogs in the networks being constructed, after adjustment by the inparalog score. The inparalog score reflects the relative similarity of a protein to its two-way best-hit ortholog and ranges from zero to one, where one indicates the maximum likelihood of orthology. The adjusted LLS for associalog is calculated by the following equation:

LLS (A′-B′) = LLS (A-B) + ln(inparalog score of A-A′) + ln(inparalog score of B-B′),

where A and B are template species proteins and A′ and B′ are their orthologs in the query species. Thus, if either orthologous relationship (A-A′ or B-B′) is weak, the LLS for the associalog between query proteins (A′-B′) is down-weighted. Many associalogs turned out to have likelihood scores below random expectation (i.e. LLS < 0) after downward adjustment by inparalog scores, and the JiffyNet system excludes all associalogs with LLS <0 from the final output networks. The edge file of the output network for a query species is downloadable from the ‘Check Status’ and ‘Results and Analysis’ pages.

**Figure 2.**
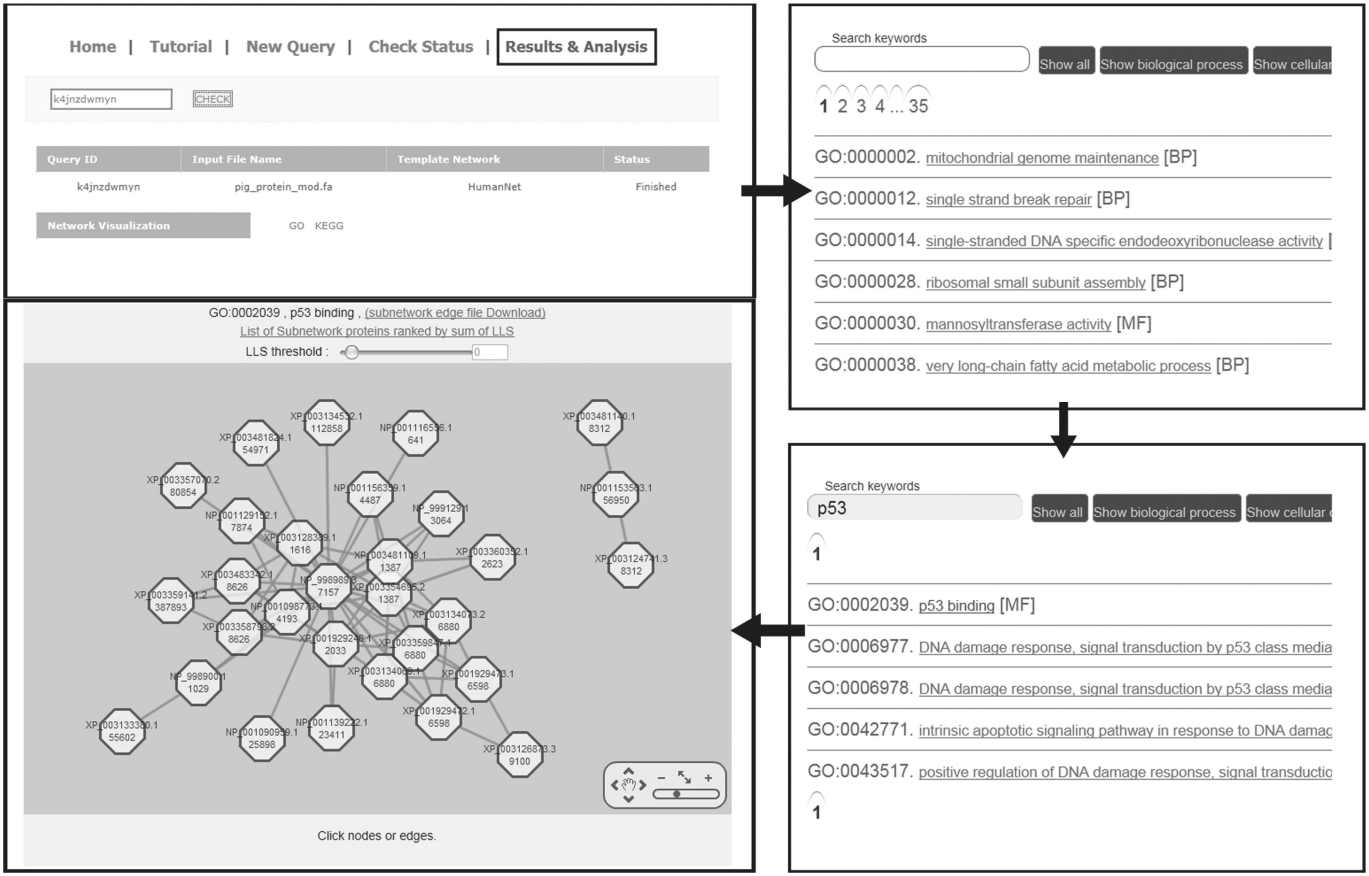
Visualization and analysis of subnetworks for molecular concepts. The ‘Results and Analysis’ page provides two links, one for a list of subnetworks by GO terms and the other by KEGG pathway terms. The linked page for GO terms lists all available terms in multiple pages (the given example has 35 pages of GO term list). These terms can be filtered for each category of GO annotation (BP for biological process, CC for cellular component or MF for molecular function). One can use keywords to search for terms of interest. For example, the keyword p53 lists up to five GO terms containing the word. Clicking linked term ‘p53 binding’ opens a new page showing a subnetwork for the molecular concept of ‘p53 binding’. Each node shows both the template and query species protein names for a pair of orthologs. The user can specify different confidence levels for edges by using the slider bar for ‘LLS threshold.’ Using viewer tools, one can zoom in or out and move the network or individual nodes. The linked page also shows a list of subnetwork proteins ranked by sum of LLS scores.

Genome-scale protein networks facilitate hypothesis generation by using network topology. Splitting an output query genome-scale network into subnetworks for individual molecular concepts, defined by annotation of template species orthologs, is perhaps an effective approach to exploring functionally uncharacterized proteins of query species. JiffyNet allows users to visualize and analyze subnetworks for each GO or KEGG term using the ‘Results and Analysis’ page (Figure 2). The network visualization function was implemented using Cytoscape web (18). Each network node exhibits both a query species protein name and a template species protein name. In each subnetwork of molecular concepts, query proteins with a large number of within-concept neighbors (i.e. hubs) may be more meaningful proteins for the molecular concept. Thus, JiffyNet also provides a list of subnetwork proteins ranked by total weighted within-concept connectivity (i.e. sum of LLS).

## BENCHMARKING

**Figure 3.**
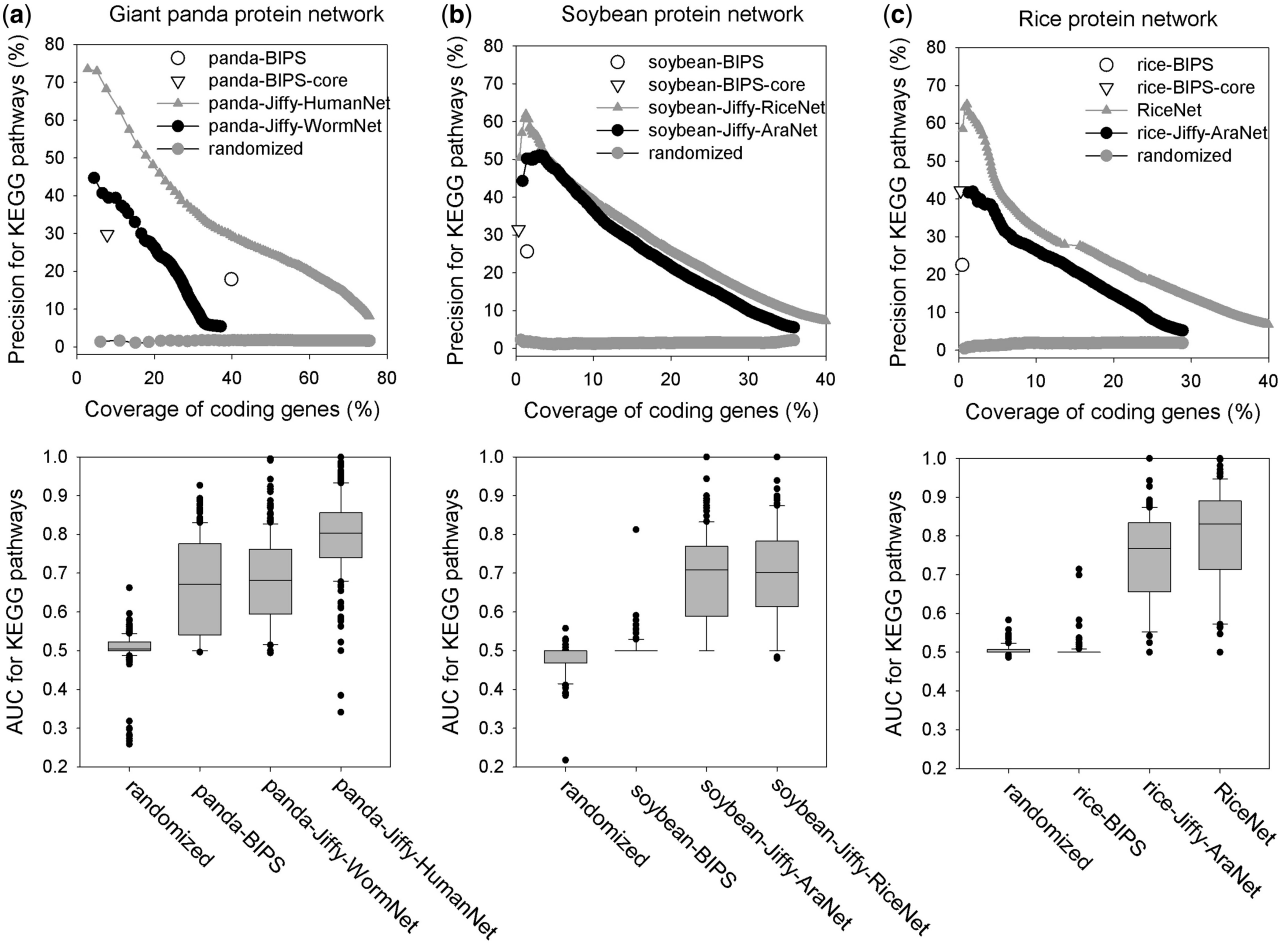
Quality assessment of protein networks for (**a**) giant panda (*A.melanoleuca*), (**b**) soybean (*G. max*) and (**c**) rice (*O. sativa*). Graphs of KEGG pathway precision as a function of coding genome coverage (upper panels) and the AUC for KEGG pathways (lower panels) are shown. In the upper panel plots, each data point represents a bin of 1000 network links, sorted by likelihood score. AUC scores are summarized as box-and-whisker plots. We created networks using JiffyNet by selecting HumanNet and WormNet as template networks for panda (panda-Jiffy-HumanNet and panda-Jiffy-WormNet, respectively), AraNet and RiceNet for soybean (soybean-Jiffy-AraNet and soybean-Jiffy-RiceNet, respectively) and AraNet for rice (rice-Jiffy-AraNet). For comparison purposes, we show networks generated for the same species using the BIPS, which creates networks on the basis of protein–protein interactions between orthologs in other species. In addition, we used BIPS to define core networks by identifying protein pairs that share GO annotations (panda-BIPS-core, soybean-BIPS-core and rice-BIPS-core). For panda, HumanNet was a more effective template than WormNet and covered a larger portion of the panda coding genome (75%). For soybean, AraNet and RiceNet were similarly effective templates, and the resultant networks covered 35–40% of coding genome. For the giant panda network, median AUC scores were 0.803 and 0.681, respectively, using the HumanNet and WormNet templates. For soybean, AUC scores were 0.708 and 0.702 using the AraNet and RiceNet templates, respectively. An AUC of 0.5 is expected if results are due to chance alone. We also compared JiffyNet results for rice (rice-Jiffy-AraNet) to a high-quality protein network constructed by integrating 24 different types of datasets (RiceNet).

The quality of the protein networks predicted by JiffyNet was assessed using (i) HumanNet and WormNet-based giant panda (*Ailuropoda melanoleuca*) protein networks (Figure 3a) and (ii) AraNet- and RiceNet-based soybean (*Glycine max*) protein networks (Figure 3b). We chose these species for assessment because their genome-scale protein network models would be proposed for the first time by JiffyNet. In addition, these species have been annotated by the KEGG pathway database, which provides independent test data for the network assessment. A total of 631 933 positive pairs and 13 543 217 negative pairs were used for assessing giant panda networks, and 463 223 positive pairs and 13 111 432 negative pairs were used for assessing soybean networks. We compared the quality of the orthology-based networks by JiffyNet and BIPS, another public web server capable of constructing orthology-based protein networks (10), using identical query protein sequence input data. The BIPS web server differs substantially from JiffyNet as described above.

Plots of KEGG pathway precision as a function of coding genome coverage demonstrate that the full-sized qualified networks constructed using JiffyNet cover a large portion of the coding genome (40–75% and 35–40%, respectively, for giant panda and soybean). In terms of precision and coverage for KEGG pathway annotations, BIPS-generated networks fall short of JiffyNet-generated networks for both panda and soybean (Figure 3a and b upper panels). As predicted, HumanNet was more effective than WormNet when using JiffyNet to construct the panda protein network. Pandas are more closely related to humans than worms, and humans harbor more panda orthologs (13 053) than worms do (6781). By contrast, when constructing the soybean protein network, AraNet and RiceNet proved to be similarly effective templates. Soybean is more closely related to *Arabidopsis* than rice, yet *Arabidopsis* has fewer soybean orthologs (20 817) than rice does (23 711). On the basis of these findings, we concluded that the effectiveness of JiffyNet is affected by not only the evolutionary distance between the template and query species but also the number of orthologs present in the template species.

For panda, the BIPS network of protein pairs sharing GO terms achieves higher precision (29.7%), but at the cost of low-coding genome coverage (7.8%) compared with full-size network (17.9% of precision for 39.9% of coding genome coverage). Notably, the higher quality panda protein network from BIPS covers only 7.8% of the coding genome, while the JiffyNet network of equal precision covers 39.6%, showing a 5-fold difference. The performance difference between JiffyNet and BIPS is even more dramatic in soybean. A higher quality BIPS network (precision is 31.5%) covers <0.35% of the soybean coding genome, while the JiffyNet one with an equal level of precision covers 15.6%, indicating almost 45-fold difference.

We also assessed how well each network recovered KEGG pathway proteins using area under Receiver Operating Characteristic curve (AUC), a convenient summary statistic for connectivity among member proteins of each pathway (19). For this analysis, we considered only 114 and 238 KEGG pathway terms with no less than five member proteins for soybean and panda, respectively. As shown in Figure 3a and b lower panels, the distribution of the JiffyNet AUC scores outperforms BIPS in both query species. The performance of JiffyNet for soybean is particularly remarkable compared with the random-level performance of BIPS. This catastrophic failure of BIPS is probably due to the lack of template protein–protein interactions for plants in current public databases.

We also validated the quality of networks generated by JiffyNet using a well-known species: rice. We previously published a high-quality protein network for rice, RiceNet, which was constructed by integrating 24 different types of datasets (15) and is a template network for JiffyNet. As shown in Figure 3c, an AraNet-based network for rice (rice-Jiffy-AraNet) is of lower quality than a RiceNet, but the two are still somewhat comparable. A total of 153 152 positive pairs and 4 240 978 negative pairs of KEGG pathway genes were used for assessing rice networks with precision and coverage analysis. For AUC analysis, we considered 113 KEGG pathway terms for rice with no less than five member proteins. The precisions for 10% coverage of the coding genome are ∼26% for rice-Jiffy-AraNet and ∼32% for RiceNet, a difference of roughly 20%. Moreover, the median AUC for rice-Jiffy-AraNet is 0.77, a level of predictability that is considered high. Similar to the results for soybean, BIPS generated networks covering no more than 0.5% of the rice coding genome. In an earlier study, the quality of an orthology-based network for rice was supported by another line of evidence: co-expression of linked proteins across cell types (15). In a study of 40 cell types, the likelihood of shared expression between linked proteins was much higher than what would be expected by chance alone in AraNet-based rice protein network (20).

## CONCLUSION

JiffyNet is a free web server for biologists who wish to construct genome-scale protein networks for their species of interest using only protein sequence data. This task is practically achievable using highly accurate genome-scale template protein networks along with a robust and sensitive orthology mapping algorithm. The procedure for network construction using JiffyNet does not require any advanced bioinformatics skills. In addition, JiffyNet provides network visualization and analysis pages for a wide variety of molecular concepts, which are often major targets for functional investigations. Therefore, JiffyNet provides a feasible solution for instant network modeling of newly sequenced species. We expect continual growth in usability of JiffyNet as more template networks are added in the future.

## FUNDING

National Research Foundation of Korea [2010-0017649, 2012M3A9B4028641, 2012M3A9C7050151]; Next-Generation BioGreen 21 Program [SSAC, PJ009029]. Funding for open access charge: National Research Grant.

*Conflict of interest statement.* None declared.
